# Increasing the activity output and optimization of automated radiosynthesis [^68^Ga]Ga-DOTATATE, [^68^Ga]Ga-Pentixafor, and [^68^Ga]Ga-FAPI-64 using two [^68^Ge]Ge/[^68^Ga]Ga iThemba generators in series

**DOI:** 10.1186/s41181-026-00426-2

**Published:** 2026-02-17

**Authors:** Ammar Alfteimi, Ulf Lützen, Alexander Helm, Michael Jüptner, Yi Zhao, Maaz Zuhayra

**Affiliations:** Department of Nuclear Medicine, Molecular Diagnostic, Imaging and Therapy, University Hospital of Schleswig-Holstein (UKSH), Camous Kiel, Karl Lennert Cancer Center North, Feld-Str. 21 (Haus L), 24105 Kiel, Germany

**Keywords:** Automated radiosynthesis, Pentixafor, DOTATATE, FAPI-46, Gallium-68

## Abstract

**Background:**

The rapidly increasing clinical demand for ^68^Ga-labelled radiopharmaceuticals continues to challenge current production capacities, particularly in high-throughput nuclear medicine departments. Although dual-generator concepts have previously been explored, all reported approaches to date have required either pre-purification, fractionated elution, or additional cartridge-based concentration steps, which add complexity and limit routine clinical implementation. In the present study, we report for the first time a fully automated, GMP-compliant synthesis protocol using two iThemba ^68^Ge/^68^Ga generators connected in series on a standard EasyOne module applicable to three clinically relevant tracers. We successfully established robust GMP production of [^68^Ga]Ga-DOTATATE, [^68^Ga]Ga-FAPI-46, and [^68^Ga]Ga-PentixaFor without any pre-purification or fractional elution. A key mechanistic finding of this work is the critical role of direct ascorbic acid addition to the reaction medium, which effectively suppresses radiolysis and metal-ion interference under high-activity conditions.

**Results:**

We established a Good Manufacturing Practice (GMP)-compliant, fully automated synthesis of [^68^Ga]Ga-DOTATATE, [^68^Ga]Ga-Pentixafor, and [^68^Ga]Ga-FAPI-46 using two ^68^Ge/^68^Ga iThemba generators connected in series, of which the older generator is replaced with a new one every 6 months. This configuration enabled elution of maximum activity of 3750 MBq and minimum activity of 2345 MBq, exceeding the elution activity achieved with a single generator by 2300 and 901 MBq respectively. The corrected yield of the labelled products was 91 ± 5% (72 ± 5% non-decay corrected). The additional activity of the labeled products obtained through the two generator configuration enables the examination of 2–4 additional patients per batch and thus resulting in significant cost savings. The direct addition of ascorbic acid to the reaction medium was essential, as it suppressed radiolysis and minimized the impact of metallic impurities. This innovation enabled reproducible labeling without pre-purification, which has not previously been demonstrated with SnO_2_-based generators.

**Conclusions:**

Dual-generator elution on the EasyOne module without modification of Trasis single-use cassettes provides a robust and scalable approach for high-yield production of ^68^Ga radiopharmaceuticals. The integration of series-connected iThemba generators with in-situ radiolysis control by ascorbic acid ensures consistent GMP-compliant synthesis of [^68^Ga]Ga-DOTATATE, [^68^Ga]Ga-Pentixafor, and [^68^Ga]Ga-FAPI-46. This method improves production efficiency, reduces costs, and expands clinical accessibility.

## Background

Positron emission tomography/computed tomography (PET/CT) using ^68^Ga-labeled radiopharmaceuticals has become an essential component of Modern nuclear medicine, offering powerful tools for tumor detection, staging, and therapy planning across a broad spectrum of malignancies (Banerjee and Pomper 2013). The clinical success of ^68^Ga tracers stems largely from the favorable physical properties of ^68^Ga, including its 68-min half-life and high positron yield, which together enable high-resolution imaging while maintaining acceptable radiation exposure for patients (Dash and Chakravarty 2019; Velikyan 2015; Zhernosekov et al. 2007).

From a radiochemistry perspective, ^68^Ga readily forms stable complexes with macrocyclic chelators, providing straightforward and reliable labeling procedures compared with ^18^F, where radiolabeling often involves multistep prosthetic-group chemistry for covalent C–F bond formation (Mu et al. 2013). Furthermore, the long-lived ^68^Ge/^68^Ga generator system enables on-demand production of ^68^Ga without the need for an on-site cyclotron, facilitating global access to ^68^Ga-based PET imaging (Zhernosekov et al. 2007).

Clinically, numerous ^68^Ga-labeled radiopharmaceuticals have demonstrated high efficacy in tumor imaging applications. Examples include [^68^Ga]Ga-DOTATATE for somatostatin receptor imaging in neuroendocrine tumors (Sandström et al. 2013), [^68^Ga]Ga-FAPI-46 for visualization of cancer-associated fibroblasts via fibroblast activation protein targeting (Da Pieve et al. 2022; Mori et al. 2023), and [^68^Ga]Ga-Pentixafor for visualization of CXCR4 expression in hematologic and solid malignancies (Herrmann et al. 2016; Kraus et al. 2022).

Despite the clinical promise of ^68^Ga-labeled tracers, their widespread adoption was initially constrained by both the limited activity output of a single ^68^Ge/^68^Ga generator, typically sufficient for only few patient doses per synthesis. Early protocols relied on pre-purification methods such as strong cation-exchange (SCX) or anion-exchange chromatography to reduce ionic impurities, lower acidity, and concentrate the eluate, thereby facilitating more efficient chelation (de Blois et al. 2011; Breeman et al. 2005; Meyer et al. 2004; Velikyan et al. 2004; Zhernosekov et al. 2007). Alternatively, fractionated elution was employed to selectively collect the central eluate fractions, recovering up to 80% of the elutable ^68^Ga activity with reduced ^68^Ge and metallic contaminants (de Blois et al. 2011; Breeman et al. 2005, 2011; Decristoforo et al. 2007; Velikyan et al. 2004). While these methods proved chemically effective, they added operational complexity, increased synthesis times, and frequently decreased apparent molar activity and effective radiochemical yield due to decay losses and additional handling steps. As a result, production efficiency and routine clinical scalability were significantly limited.

Subsequent advances in generator technology have transformed the field. Improvements in column chemistry have reduced ^68^Ge breakthrough to consistently below the European Pharmacopoeia limit of 0.001% (Tworowska et al. 2016), enabling SCX-free and fully automated syntheses that now represent the state of the art. Several groups have reported simplified workflows achieving pharmacopoeia-compliant preparations of [^68^Ga]Ga-DOTATATE (Shukla et al. 2012; Tworowska et al. 2016), [^68^Ga]Ga-FAPI-46 (Alfteimi et al. 2022), and [^68^Ga]Ga-Pentixafor (Costes et al. 2023) without pre-purification.

Currently, multiple commercial ^68^Ge/^68^Ga generators are available for clinical use, differing in column matrix, eluent composition, and impurity profiles. TiO_2_-based systems such as GalliaPharm^®^ (Eckert & Ziegler, Germany) are widely regarded as the clinical reference standard, offering reproducible elution with approximately 70% efficiency in 7 mL of 0.1 M HCl, stable ^68^Ge breakthrough below 0.001% over at least 12 months, and metallic impurities typically between 1 and 10 ppm (Amor-Coarasa et al. 2016; Romero et al. 2020; Zhernosekov et al. 2007). By comparison, SnO_2_-based iThemba generators (iThemba LABS, South Africa), eluted with 0.6 M HCl, achieve approximately 80% elution efficiency but exhibit higher and more variable metallic impurities, often between 1 and 20 ppm, complicating chelation chemistry despite consistently acceptable ^68^Ge breakthrough levels below 0.001% (Sriprapa et al. 2023, 2024; Sudbrock et al. 2014; Asti et al. 2008). Consequently, pre-conditioning or fractionated elution has traditionally been required when using iThemba generators, limiting their routine applicability compared with TiO_2_-based systems.

To address the intrinsic limitation of activity output from a single generator, dual-generator configurations have been explored to scale production for high-throughput clinical settings. Using TiO_2_-based systems, Brusa et al. (2025) scaled up [^68^Ga]Ga-FAPI-46 production on a Modular-Lab PharmTracer platform with two GalliaPharm^®^ generators and an intermediate cation-exchange step, achieving a decay-corrected yield of 91.1% and radiochemical purity above 99.8%, with batch stability maintained using sodium ascorbate. Similarly, van Brandwijk et al. (2024) employed two GalliaPharm^®^ generators for automated [^68^Ga]Ga-PSMA-11 synthesis using the Locametz^®^ kit, obtaining radiochemical yields of 96–101% and purities between 98.4 and 99.3% without pre-purification. Durieux et al. (2023) demonstrated that double fractional elution from two GalliaPharm^®^ generators enabled [^68^Ga]Ga-PSMA-11 synthesis with mean labeling yields of 97.5 ± 1.9%, radiochemical purity around 96%, and overall radiosynthesis yields of 58%, outperforming double elution with pre-purification, which produced lower and less consistent yields.

Beyond TiO_2_-based systems, the feasibility of scaling ^68^Ga production from SnO_2_-based iThemba generators has been demonstrated using two distinct dual-generator approaches. Rossouw and Breeman (2012) first employed fractionated elution from two iThemba generators to isolate the most concentrated central fractions, recovering approximately 95% of the activity in 4 mL and enabling direct [^68^Ga]Ga-DOTATATE synthesis with decay-corrected radiochemical yields above 89% following C18 purification. Building on this work, Kvaternik et al. (2016) combined eluates from two iThemba generators and passed them through a PS-H^+^ cation-exchange cartridge, achieving quantitative trapping of ^68^Ga up to 98% of the theoretical activity and labeling yields above 40% (non–decay corrected) for [^68^Ga]Ga-DOTANOC. While both methods demonstrated the technical feasibility of scaling ^68^Ga production from iThemba generators, each required either fractionation or cartridge-based trapping, adding operational complexity and limiting routine use.

To our knowledge, no study has yet reported the direct, fully automated synthesis of [^68^Ga]Ga-DOTATATE, [^68^Ga]Ga-FAPI-46, or [^68^Ga]Ga-Pentixafor using eluates from two iThemba generators connected in series without fractionation elution or pre-purification. The aim of the present study was to demonstrate that Good Manufacturing Practice (GMP)-compliant production of [^68^Ga]Ga-DOTATATE, [68Ga]Ga-FAPI-46, and [68Ga]Ga-Pentixafor can be achieved using the EasyOne synthesis module without fractional elution and pre-purification of [^68^Ga]GaCl_3_ obtained from two ^68^Ge/^68^Ga iThemba generators connected in series. A key factor enabling the success of this approach was the addition of ascorbic acid to the reaction medium, which effectively suppressed radiolysis and minimized interference from metallic impurities, thereby eliminating the need for pre-purification. This strategy offers several advantages, including reduced consumption of costly non-radioactive precursors, decreased radiation exposure to radiochemists by minimizing the number of syntheses per day, and the capacity to produce additional patient doses per batch, thereby improving cost efficiency. Over more than 3 years of clinical implementation, this dual-generator strategy has consistently yielded robust, high-purity, and time-efficient production of [^68^Ga]Ga-DOTATATE, [^68^Ga]Ga-FAPI-46, and [^68^Ga]Ga-Pentixafor, greatly facilitating their routine clinical use.

## Methods and materials

All single-use cassettes and reagent kits for the radio synthesis were sterile and manufactured under GMP. Ascorbic acid (manufactured under GMP) was purchased from ABX Advanced Biochemicals (Radeberg, Germany). The DOTATATE-peptide were purchased in 50 µg single-use vials as a GMP product from ABX. The FAPI-peptide was purchased in 50 µg single-use vials as a GMP product from SOFIE Biosciences. Pentixafor was purchased from MedChemExpress (MCE; Monmouth Junction, NJ, USA). in Other chemicals were purchased from commercial sources and were used without further purification. two ^68^Ge/^68^Ga generators (iThemba generator, 50 mCi Somerset West, South Africa) were placed in series (6-month-old ^68^Ge/^68^Ga generator and new ^68^Ge/^68^Ga generator).

### Preparation of the ascorbic acid solution

3 mg of Ascorbic Acid powder were put into a clean and sterilized vial (2 ml) and 400 µl of Acetate buffer (1.5 M) were added to dissolve the powder. The Vial was closed with a sterile septum cap, then put in an ultrasonic water bath until the powder is dissolved completely.

### ^68^Ge/^68^Ga generators

**Fig. 1:**
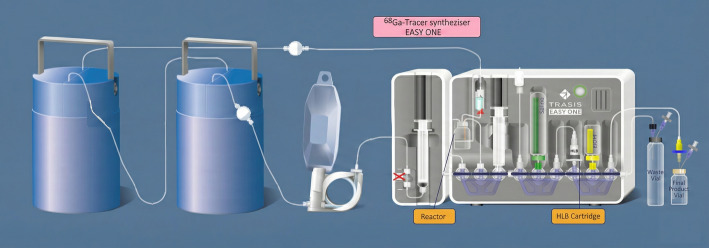
Scheme of automated synthesizer of [^68^Ga]Ga-DOTATATE, [^68^Ga]Ga-Pentixafor, and [^68^Ga]Ga-FAPI-64 (EasyOne; Trasis)

Two 50 mCi (1.85 GBq) iThemba generators were connected in series. The sterile ultrapure HCl solution was connected to the older generator inlet port and the outlet was then connected to the newer generator inlet port. The outlet was connected to the inlet port of the cassette (Fig. 1). These generators were eluted daily with 6 mL of 0.6 M HCl prior to labeling in order to avoid the accumulation of free long-life ^68^Ge ions and metal impurities in the final product. The older generator is replaced every 6 months.

### Synthesis of [^68^Ga]Ga-FAPI-46

The fully automated synthesis of [^68^Ga]Ga-FAPI-46 was performed with Trasis EasyOne synthesizer (operated by the software Trasis Supervision^®^ (Trasis, Ans, Belgium) shown in Fig. 1 we used the single-use cassette and reagent kit supplied by Trasis for the synthesis of ^68^Ga peptides. The reagent kit contains an acetate buffer syringe as well as saline and ethanol vials. The disposable cassette includes a 10 ml reactor, one 10 ml syringe and spikes for connection with two prefilled vials as well as an Oasis HLB cartridge. The labelling process involved preheating the reaction vessel containing the reaction mixture (98 °C), adding the [^68^Ga]GaCl_3_ solution and continued heating for 5 min as follows: The reaction mixture containing 3 ml of acetate buffer solution, 400 µl of the Ascorbic acid solution and 50 µg of FAPI-46 was loaded into the reaction vial. [^68^Ga]GaCl_3_ was then eluted into the reaction vial from the two ^68^Ge/^68^Ga generators with 6 ml of 0.6 M HCl. For purification, the crude reaction mixture was passed through a HLB Sep-Pak cartridge to trap the labelled peptide by retaining the [^68^Ga]Ga-FAPI-46 while 68Ga impurities passed through the HLB Sep-Pak cartridge into a waste vial. The HLB Sep-Pak cartridge was then washed with 5 ml of 0.9% aq. NaCl to remove most of the possibly remaining free [^68^Ga]GaCl_3_ as well as buffer and traces of ^68^Ge breakthrough. The [^68^Ga]Ga-FAPI-46 was then eluted with 1 mL of ethanol, passed through a 0.22 μm filter, and collected in the product vial, which contained a solution of 0.5 mg of vitamin C in 1 mL of saline, before being further diluted with 10 mL of 0.9% aq. NaCl.

### Synthesis of [^68^Ga]Ga-Pentixafor

The fully automated synthesis of [^68^Ga]Ga-Pentixafor was also performed with Trasis EasyOne synthesizer. The reaction mixture containing 3 ml of Acetate buffer solution (1.5 M), 400 µl of the Ascorbic acid solution and 50 µg of the Pentixafor precursor was loaded into the reaction vial. Then the [^68^Ga]GaCl_3_ solution was directly eluted with 6 ml of 0.6 M HCl into the reaction vial from the two generators and heated 120 °C for 1.5 min followed at 95 °C for 8.5 min. The purification of the crude reaction mixture and formulation of [^68^Ga]Ga-Pentixafor was carried out as described above for [^68^Ga]Ga-FAPI-46.

### Synthesis of [^68^Ga]Ga-DOTATATE

[^68^Ga]Ga-DOTATATE was synthesized using Trasis EasyOne synthesizer as follows: The reaction mixture containing 3 ml of Acetate buffer solution (1.5 M) 1.5 mg of ascorbic acid and 50 µg of DOTATATE were added into the reactor vial. The [^68^Ga]GaCl_3_ was then eluted from the two ^68^Ge/^68^Ga generators with 6 ml of 0.6 M HCl into the reaction vial at 120 °C. The following synthesis steps are the same as described above.

### Quality control

Quality control (QC) procedures for [^68^Ga]Ga-FAPI-46, [^68^Ga]Ga-Pentixafor and [^68^Ga]Ga-DOTATATE, based upon the current requirements for radiopharmaceuticals laid out in the European Pharmacopoeia for preparation of radiopharmaceuticals, quality control release criteria, are summarized below (Table 1).

**Table 1:** Summary of the product specifications for [^68^Ga]Ga-DOTATATE, [^68^Ga]Ga-FAPI-64 and [^68^Ga]Ga-Pentixafor n = 3

QC test	Release criteria	[^68^Ga]Ga-DOTATATE	[^68^Ga]Ga-FAPI-64	[^68^Ga]Ga-Pentixafor
Yield %	N/A	90.95%	90,32%	91,06%
Visual inspection	Clear, colorless	Clear, colorless	Clear, colorless	Clear, colorless
Radio chemical identity	RRT^*^ = 0.9–1.1	1.01	1.01	1.01
Radio chemical purity	> 95%	> 99,7%	> 99,3%	> 99,4%
Dose pH	4.0–8.0	4.8	4.8	4.8
Sterile Filter Integrity Test	> 3,2 bar	> 3,2 bar	> 3,2 bar	> 3,2 bar
Radionuclidic Identity (t_1/2_)	62–74	67	67	67
Radionuclidic identity (γ-spectrum)	≥ 99.9%	> 99.9%	> 99.9%	> 99.9%
Radionuclidic purity (^68^Ge breakthrough)	≤ 0.001%	< 0.001%	< 0.001%	< 0.001%
Endotoxin analysis	≤ 175 EU/ml	< 5.0 EU/ml	< 5.0 EU/ml	< 5.0 EU/ml
Sterility testing	No colony growth out to 14 days	Pass	Pass	Pass

*: Relative Retention Time

### Radio chemical identity and purity

#### HPLC analyses

The radio chemical identity (Table 1) were conducted using a System Agilent 1269 HPLC System (Agilent, USA) equipped with an UV detector set to 225 nm and Ginastar gamma raγ detector ((Elysia-raytest GmbH, Germany)). As the stationary phase a Chromolith HR RP-18 100 × 3 mm HPLC-column (Merck KGaA, Germany) was used. The mobile phase consisted of a gradient of solvents A (H_2_O containing0.1% TFA) and B (acetonitrile containing 0.1% TFA; 0–8 min 0–100% B) The flow rate was 1.0 mL/min.

The retention time of [^68^Ga]Ga-FAPI-46, [^68^Ga]Ga-Pentixafor and [^68^Ga]Ga-DOTATATE were compared to that of the [^67^Ga]Ga-Peptide reference standard and had to be within ± 10% of the standard relative retention time (RRT).

#### Thin layer chromatography analysis (TLC)

The radio chemical purity was conducted using an Elysia-raytest linear analyser detector RITA (Elysia-raytest GmbH, Germany).

- *Stationary phase A*: Tec-Control blue, (Biodex Medical Systems, USA); mobile phase A: 0.1 M aq. sodium citrate.
- *Stationary phase B*: iTLC-SG (Agilent Technologies Inc; USA); mobile phase B: 1/1 mixture of 0.4 M aq. ammonium acetate/ethanol).

Radio chemical purity had to be > 95%. All doses met these specifications (Table 1).

### Radionuclidic identity and purity

The radionuclidic identity and purity of the ^68^GaCl_3_-eluate and the final products were determined by gamma spectroscopy using a High Purity Germanium (HPGe) radiation detector (Ortec, Oak Ridge, TN, USA). The results are summarized in Tables 1 and 2. For the determination of the radionuclide identity 10 µl sample of 1:50 dilution the product (5–30 kBq) was analyzed. The measured gamma photons must have energies of 0.511 MeV and 1.077 MeV. Afterward, the half-life was determined under identical geometric conditions at five time points, with 20-min intervals between measurements. The calculated half-life must be 62 min to 74 min.

**Table 2:** Quality control of [^68^Ga]GaCl_3_ solution prior to radiolabelling according to Ph. Eur. Monograph 2464

QC Test	Acceptance Criteria (Ph. Eur. 2464)	Method	Result
Appearance	Clear, colorless solution	Visual inspection	Clear, colorless
pH	1.0–1.5	pH indicator strip	1.2
Radionuclidic identity (γ-spectrum)	Characteristic γ-emission at 511 keV	HPGe γ-spectrometry	< 99.9%
Half-life (t^1/2^)	62–74 min	Repeated activity measurements	67 min
Radionuclidic purity (^68^Ge breakthrough)	≤ 0.001% of total activity at time of use	HPGe γ-spectrometry after 48 h	< 0.001%

For determining the radionuclide purity, the same sample was measured after 48 in the gamma spectrometer (measuring time 3 h) under the same geometric conditions for determining the activity of 68Ga (resulting from 68Ge) and other radionuclidic impurities with a half-life longer than 5 h. The activity after 48 h must be < 0.001% of the eluted activity from the generator.

### Sterile filter integrity test

The sterile filter (with needle still attached) was connected to a nitrogen supply via a regulator. The needle was submerged in water and the nitrogen pressure was gradually increased. If the pressure was raised above the filter acceptance pressure (3.2 bars) without seeing a stream of bubbles, the filter was considered intact (Table 1).

### Dose pH

The pH of a small aliquot of the [^68^Ga]Ga-peptide solution was assessed using Macherey Nagel® pH 2.0–9.0 non-bleeding pH indicator strips. The colorimetric response was initially evaluated by visual inspection and subsequently analysed using a QUANTOFIX Relax test strip reader (Macherey Nagel®) for instrument-assisted optical assessment. As summarized in Table 1, the measured pH values ranged between 4.7 and 5.0.

### Metal ion analysis

The concentration of metal ions of iron, zinc and copper in the eluted activity solution was measured using indicator strips and analyzed with QUANTOFIX Relax test strip reader (Macherey Nagel®). The measured concentrations were below the detection limit of 5 ppm.

### Endotoxin analysis

The endotoxin content in the synthetic samples of [^68^Ga]Ga-peptide was analyzed using a Endosafe® nexgen-PTS™, Charles River. Doses had to contain ≤ 175 Endotoxin Units (EU) /mL to be deemed acceptable. Limulus-amoebocyte-lysate (LAL) test for bacterial endotoxin resulted < 17.5 EU/ml in all of the synthetic samples (Table 1).

### Sterility testing

Sterility testing was performed according to section 2.6.1 of the European Pharmacopoeia. Pharmacopoeia reference microorganisms used for growth promotion tests on liquid thioglycolate and soybean casein peptone medium according to Ph. Eur. 2.6.1 served as positive controls. The suitability of the media was confirmed by manufacturer’s batch certificates and routine laboratory controls.

Fluid thioglycolate media (FTM) plates and soybean casein digest agar media (SCDM) plates were treated with samples of [^68^Ga]Ga-Peptide. FTM plates were used to test for anaerobes, aerobes and micro aerophiles whilst SCDM plates were used to test for non-fastidious and fastidious microorganisms. [^68^Ga]Ga-peptide treated plates were incubated along with positive and negative controls for 14 days. FTM plates were incubated at 32 °C and SCDM plates were incubated at 22 °C according to the current USP guidelines. Plates were visually inspected on the 3rd, the 8th and the 14th day of the test and compared to the positive and negative standards. Positive standards had to show growth (turbidity) on the plates and [^68^Ga]Ga-Peptide negative controls had to have no culture growth after 14 days to be indicative of sterility. All samples met the sterility specifications (Table 1).

## Results

**Fig. 2:**
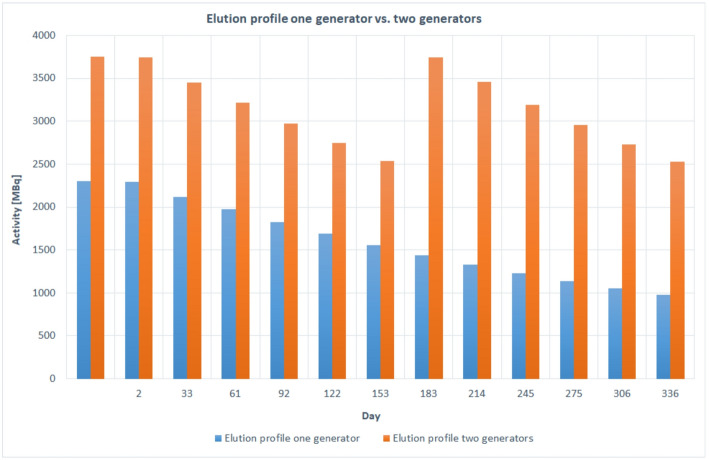
Elution profiles comparison for one- and two generator systems in 1 year

**Fig. 3:**
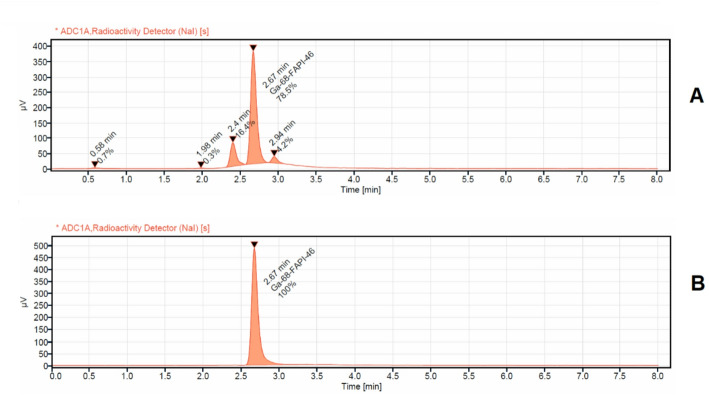
Radio chemical purity detection of [^68^Ga]Ga-FAPI-46 analized via HPLC with UV detector at 225 nm and radioactivity detector (NaI). **A**: for [^68^Ga]Ga-FAPI-46 synthesized using the same cassette and chemistry kit for labelling [^68^Ga]Ga-PSMA-11 (with addition of 400 µL of 1.5 M acetate buffer, no addition of ascorbic acid); **B**: the same as **A**, but with addition of 3 mg of ascorbic acid dissolved in 400 µL of 1.5 M acetate buffer into the buffer systems

**Fig. 4:**
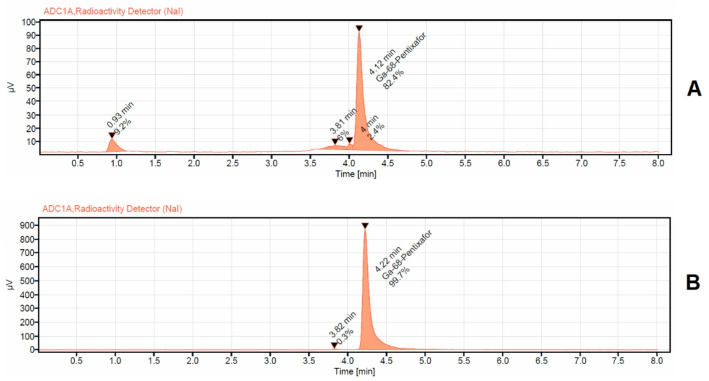
Radio chemical purity detection of [^68^Ga]Ga-Pentixafor analized via HPLC with UV detector at 225 nm and radioactivity detector (NaI). **A**: for [^68^Ga]Ga-Pentixafor synthesized using the same cassette and chemistry kit for labelling[68Ga]Ga-PSMA-11(with addition of 400 µL of 1.5 M acetate buffer, no addition of ascorbic acid); **B**: the same as **A**, but with addition of 3 mg of ascorbic acid dissolved in 400 µL of 1.5 M acetate buffer into the buffer systems

**Fig. 5:**
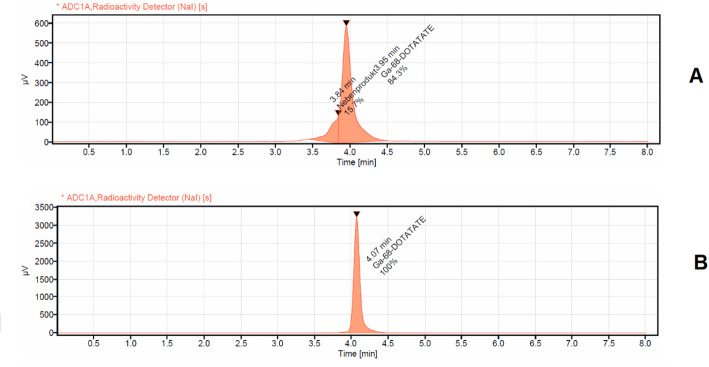
Radio chemical purity detection of [^68^Ga]Ga-DOTATATE analized via HPLC with UV detector at 225 nm and radioactivity detector (NaI). **A**: for [^68^Ga]Ga-DOTATATE synthesized using the same cassette and chemistry kit for labelling [^68^Ga]Ga-PSMA-11(no addition of ascorbic acid); **B**: the same as **A**, but with addition of 1,5 mg of ascorbic acid into the buffer systems

The elution profile of two generators versus one generator for 1 year is illustrated in Fig. 2. The activity eluted from the two iThemba generators ranged between 3750 MBq and 2345 MBq while the activity eluted from one generator was between 2300 MBq and 903 MBq. Thus, the additional obtained activity by the two generator configuration is maximum 2300 MBq and minimum 911 MBq respectively. The corrected yield of the labelled product was 91 ± 5% (72 ± 5% non-decay corrected). Accordingly, the additional obtained activity of the labelled products with two generators was in the range between 1656 MBq and 656 MBq higher than with a single generator. The radiochemical purity was > 99%. The radiochemical purity analyses for the first conducted syntheses [^68^Ga]Ga-Pentixafor, [^68^Ga]Ga-DOTATATE, and [^68^Ga]Ga-FAPI-46 synthesized using two [^68^Ge]/[^68^Ga] generators connected in series and the same chemistry kits and cassette, normally used for DOTATATE labeling, showed in all cases an insufficient radiochemical purity of < 90% (Figs. 3A, 4A and 5A).

The generator elution protocol was the same as with the standard single-generator setup. Connecting two ^68^Ge/^68^Ga generators in series resulted in only a marginal increase in elution time, which did not affect the overall synthesis workflow. Also no pressure-related alarms or system errors were observed during any of the elutions. The Trasis synthesis module is equipped with built-in safety mechanisms that automatically interrupt the process if predefined pressure limits are exceeded. Such interruptions were not encountered at any stage of the study.

In the case of [^68^Ga]Ga-FAPI-46, the representative radio-HPLC chromatogram (Fig. 3A) displays a prominent central peak at a retention time of 2.67 min, corresponding to the main product and accounting for approximately 80% of the total radioactivity. Two additional peaks—eluting at 2.41 and 2.98 min together comprise around 20% of the total radioactivity, indicating the presence of radiochemical impurities, likely arising from side reactions or partial degradation of the compound. For [^68^Ga]Ga-Pentixafor, the radio-HPLC chromatogram (Fig. 4A) also shows a strong central peak at 4.12 min, representing the primary compound and constituting about 82% of the total radioactivity. A minor shoulder is observed adjacent to the main peak at 3.81 min, contributing approximately 4% of the total activity. Additionally, an early-eluting peak at 1.0-min accounts for around 9% of the activity, suggesting the presence of both early-eluting impurities and poorly resolved species. Similarly, the chromatogram of [^68^Ga]Ga-DOTATATE (Fig. 5A) reveals a main peak at a retention time of 4.0 min, corresponding to roughly 84% of the total radioactivity. A broad, unresolved shoulder near the main peak at 3.84 min contributes approximately 16% of the total activity, further highlighting the insufficient radiochemical purity under the given synthesis conditions.

**Fig. 6:**
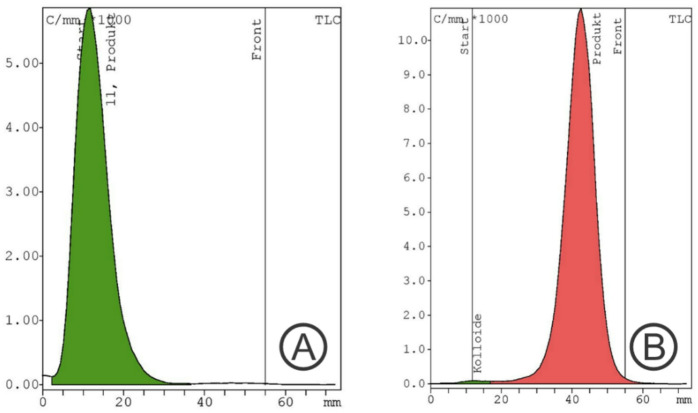
Radiochemical purity analyzed with radio TLC scanner (example: [^68^Ga]Ga-FAPI-46)

In the subsequent experiments, the addition of ascorbic acid to the reaction mixture prior to the start of the labeling process led to a marked improvement in the radiochemical purity of all three compounds: [^68^Ga]Ga-DOTATATE, [^68^Ga]Ga-FAPI-46, and [^68^Ga]Ga-Pentixafor. Specifically, the incorporation of 1.5 mg of ascorbic acid with [^68^Ga]Ga-DOTATATE, and 3 mg of ascorbic acid dissolved in 400 µL of 1.5 M acetate buffer with both [^68^Ga]Ga-FAPI-46 and [^68^Ga]Ga-Pentixafor, resulted in radiochemical purities exceeding 99.5% for all tracers as illustrated in a representative radio HPLC chromatogram in Figs. 3b, 4b and 5b and in the TLC analysis displayed in Fig. 6. Repeated analyzes up to 3 h after synthesis showed that the radio chemical purity was still > 95%. These findings highlight the crucial role of ascorbic acid as a stabilizing agent, particularly under synthesis conditions utilizing generator-produced [^68^Ga] without pre-purification.

Each synthesis provided sufficient activity for imaging up to five patients, and re-elution after 2 h recovered more than 83% of the initial generator yield, enabling efficient high-throughput production under routine clinical conditions.

## Discussion

The rapidly growing clinical demand for ^68^Ga-labeled radiopharmaceuticals, particularly in oncology, is driven by the expanding range of diagnostic applications and the global accessibility of ^68^Ge/^68^Ga generators. These generators enable on-site ^68^Ga production without the need for a cyclotron. Nevertheless, the modest activity output of a single generator, typically sufficient for one to three patient doses per synthesis, represents a critical limitation in high-throughput clinical environments.

To address this challenge, we developed and validated a dual-generator serial elution protocol using two iThemba ^68^Ge/^68^Ga generators (1.85 GBq each). In this configuration, sterile 0.6 M HCl passes sequentially through the older generator and then the newer one, eluting directly into the synthesis cassette in a total volume of 6 mL. The eluate is immediately combined with precursor solution containing buffer and ascorbic acid and heated, entirely eliminating the need for pre-purification or fractionated elution. Over 3 years of clinical implementation, this strategy reliably produced sufficient activity for up to six patient doses per batch while consistently fulfilling all Good Manufacturing Practice (GMP) requirements for sterility, stability, and radiochemical purity across multiple tracers, including [^68^Ga]Ga-PSMA-11, [^68^Ga]Ga-DOTATATE, [^68^Ga]Ga-FAPI-46, and [^68^Ga]Ga-Pentixafor. To our knowledge, this is the first automated, GMP-compliant dual-generator protocol applied to multiple tracers without the need for pre-purification or elution fractionation.

A critical factor in achieving high and reproducible radiochemical yields under dual-generator conditions is precursor mass. Under single-generator operation, 20–25 µg of DOTATATE ensures a large ligand excess, maintaining pseudo–first-order kinetics and enabling rapid, nearly quantitative Ga^3+^ incorporation (Sudbrock et al. 2014; Zhernosekov et al. 2007; Velikyan et al. 2004). However, eluates from two generators have increased concentrations of competing metal ions in the system. This circumstance diminishes the kinetic advantage of ligand excess, increases the risk of incomplete chelation, promotes Ga(OH)_3_ precipitation, and introduces batch-to-batch variability. Similar observations were previously reported by Rossouw and Breeman (2012), who found that precursor quantities of approximately 50 µg were required to maintain reproducibility under dual-generator conditions. Consistent with these findings, our results demonstrate that at 25 µg precursor mass, decay-corrected radiochemical yields varied substantially (10–90% for DOTATATE and below 30% for FAPI-46 and Pentixafor), with radiochemical purity frequently dropping below the European Pharmacopoeia threshold of 95%. Increasing the precursor mass to 50 µg for all tracers, together with the addition of ascorbic acid prior to labeling, consistently restored a ligand-excess kinetic regime, yielding approximately 92% decay-corrected radiochemical yield and radiochemical purity above 98%. Mechanistically, this strategy accelerates Ga–DOTA complexation, suppresses colloid formation, and mitigates the effects of radiolysis and trace-metal interference.

Potential concerns regarding the complexation of tetravalent impurities such as ^68^Ge by DOTA ligands warrant careful consideration. However, coordination chemistry studies have demonstrated that DOTA strongly prefers di- and trivalent cations, while tetravalent ions such as Zr^4+^ or Hf^4+^ do not effectively compete under physiological conditions (Meyer et al. 2004; Breeman et al. 2003; Heppeler et al. 1999). Furthermore, the radiochemical implications of ^68^Ge breakthrough in generator-based ^68^Ga production have been thoroughly investigated, particularly with respect to its potential impact on patient safety. Mechanistically, solid-phase extraction using reversed-phase C18 sorbents efficiently captures unchelated metal ions via hydrophobic interactions with the alkyl-bonded stationary phase, achieving more than a 100-fold reduction in ^68^Ge content and ensuring compliance with the European Pharmacopoeia specification of 0.001% (Decristoforo et al. 2007). Similarly, hydrophilic–lipophilic balanced (HLB) resins operate through a mixed-mode retention mechanism combining hydrophobic partitioning and polar interactions, enabling efficient removal of unbound ^68^Ga species and the reduction of trace ^68^Ge impurities during post-processing (Ammour et al. 2025; Brom et al. 2016; Karacay et al. 2011). Although a universal quantitative reduction factor for HLB-based purification has not yet been established, our findings demonstrate that its incorporation into the automated synthesis workflow resulted in a more than tenfold decrease in ^68^Ge content in the final product vial, even under dual-generator operational conditions. Consistent daily generator elution, combined with systematic generator replacement at 6-month intervals, further maintained ^68^Ge breakthrough well below European Pharmacopoeia limits while simultaneously minimizing the accumulation of metallic contaminants.

Importantly, dosimetric analyses by Velikyan et al. (2013) have demonstrated that the current European Pharmacopoeia limit is highly conservative, suggesting that permissible ^68^Ge impurity levels in ^68^Ga eluates could be safely increased by more than 100-fold without any measurable impact on patient dosimetry. Taken together, our findings suggest that the dual-generator strategy described here may be broadly applicable across different commercially available ^68^Ge/^68^Ga generator platforms. The consistently low ^68^Ge breakthrough values observed in this study, combined with stable radiochemical performance under high-activity conditions, indicate that this approach provides a robust and radiologically safe framework for routine ^68^Ga-radiopharmaceutical production in clinical settings.

In summary, the integration of serial dual-generator elution with precursor mass optimization and pre-labeling addition of ascorbic acid provides a mechanistically rational and GMP-compliant solution to the major challenges of high-activity ^68^Ga production. This approach ensures reproducible radiolabeling, controls radiolysis, and preserves favourable chelator-to-metal stoichiometry while simplifying the synthesis workflow. Its adaptability, scalability, and demonstrated clinical reliability make it a compelling strategy for expanding access to ^68^Ga-labeled radiopharmaceuticals in high-demand clinical settings. Furthermore, all radiolabeled products demonstrated radiochemical purities exceeding 95% after 3 h of incubation at room temperature. Thereby confirming the robustness and stability of the process under routine clinical conditions.

## Conclusion

This study presents, for the first time, a fully automated, GMP-compliant dual-generator serial elution protocol for the production of [^68^Ga]Ga-DOTATATE, [^68^Ga]Ga-FAPI-46, and [^68^Ga]Ga-Pentixafor without any need for pre-purification or eluate fractionation. The direct addition of ascorbic acid to the reaction mixture proved essential under high-activity conditions, ensuring radiochemical purity consistently above 99.5% and tracer stability for at least 3 h post-synthesis. Optimization of precursor mass to 50 µg re-established a ligand-excess kinetic regime even with dual-generator operation, achieving reproducible decay-corrected radiochemical yields exceeding 90% across all tracers. Each production run provided sufficient activity for up to six patient doses, while short elution intervals facilitated rapid recovery of generator capacity, thereby enhancing operational efficiency.

Implemented routinely in our clinical radiopharmacy over more than 3 years, this protocol has demonstrated excellent robustness, reproducibility, and full compliance with European Pharmacopoeia quality specifications. These findings underscore the critical role of antioxidant stabilization in mitigating radiolysis and metal-ion interference during high-activity ^68^Ga labeling and establish a scalable, clinically validated approach suitable for broader application across diverse ^68^Ge/^68^Ga generator platforms. This strategy offers a practical solution to the increasing global demand for ^68^Ga-labeled radiopharmaceuticals in molecular imaging.

## Data Availability

All data generated or analyzed during this study are included in this published article.

## Abbreviations

[^68^Ga]Ga-DOTATATE: Gallium-68-labelled DOTA-Tyr^3^-octreotate
[^68^Ga]Ga-FAPI-46: Gallium-68-labelled fibroblast activation protein inhibitor-46
[^68^Ga]Ga-Pentixafor: Gallium-68-labelled CXCR4 ligand
^68^Ge/^68^Ga generator: Germanium-68/gallium-68 generator system
CXCR4: C-X-C chemokine receptor type 4
DPP4: Dipeptidyl peptidase 4
FAP: Fibroblast activation protein
GMP: Good manufacturing practice
HLB: Hydrophilic-lipophilic balance
HPLC: High performance liquid chromatography
PET: Positron emission tomography
PET/CT: Positron emission tomography/computed tomography
Ph. Eur.: European Pharmacopoeia
ppm: Parts per million
QC: Quality control
RCY: Radiochemical yield
RCP: Radiochemical purity
SCX: Strong cation exchange
SnO_2_: Tin dioxide (generator column matrix)
TiO_2_: Titanium dioxide (generator column matrix)
TLC: Thin-layer chromatography
